# Supplementary figures and images for: ATP13A4 Upregulation Drives the Elevated Polyamine Transport System in the Breast Cancer Cell Line MCF7
Source: Biomolecules. 2023 May 31;13(6):918. doi: 10.3390/biom13060918 (PMC10296708; doi:10.3390/biom13060918)

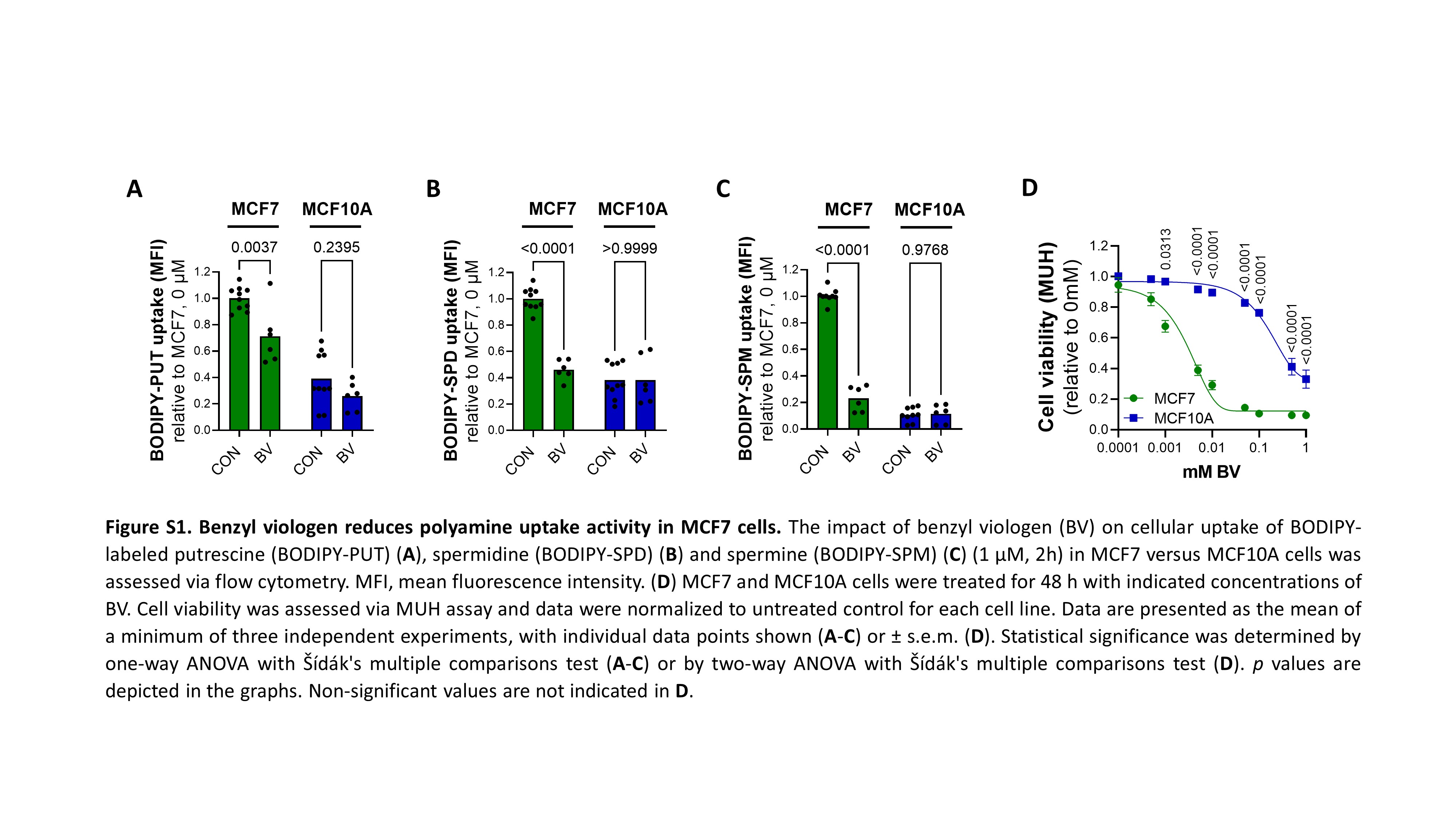

Supplement: Supplementary file 1 [file biomolecules-13-00918-s001.zip › Figure S1.JPG]

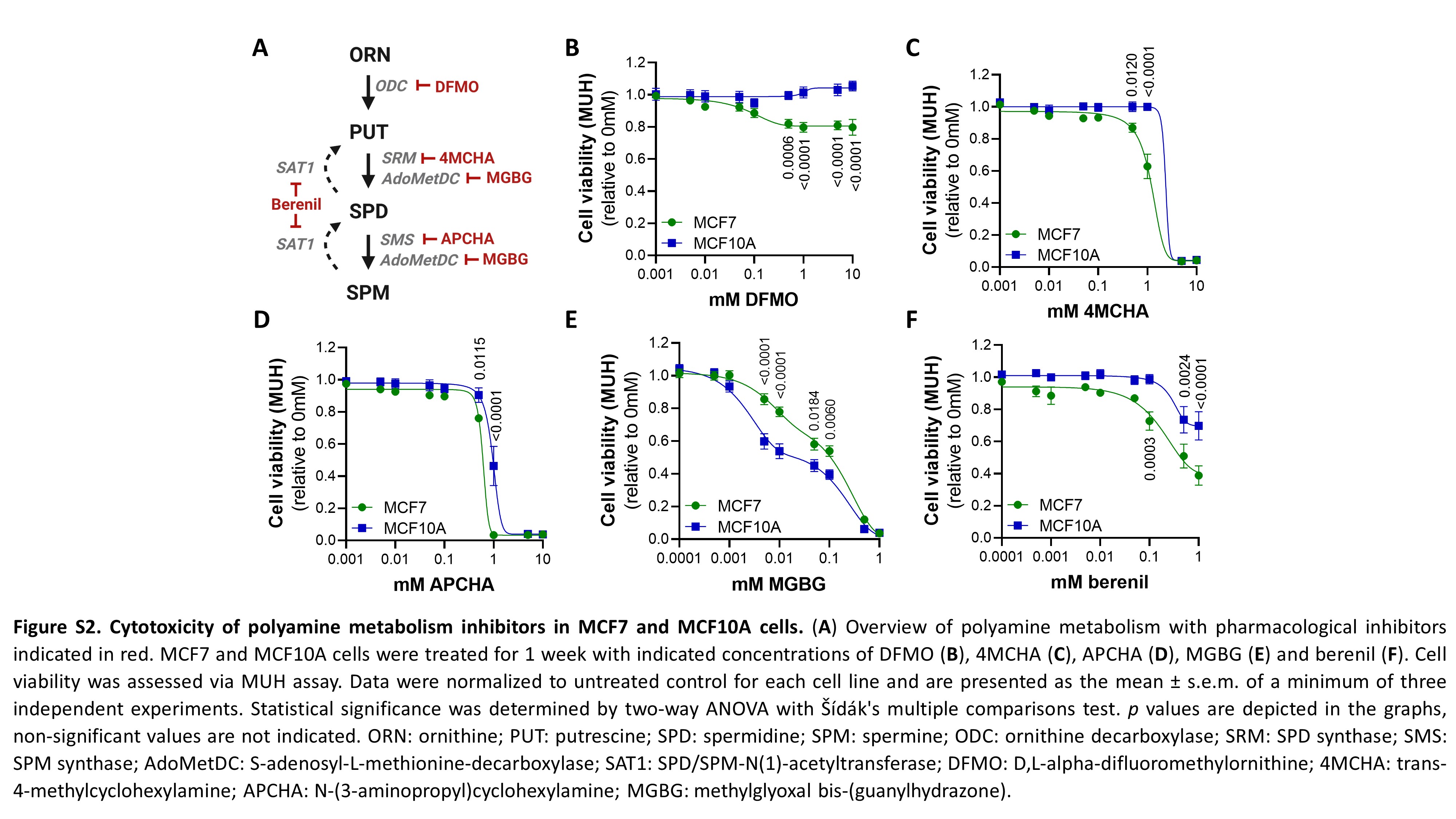

Supplement: Supplementary file 1 [file biomolecules-13-00918-s001.zip › Figure S2.JPG]

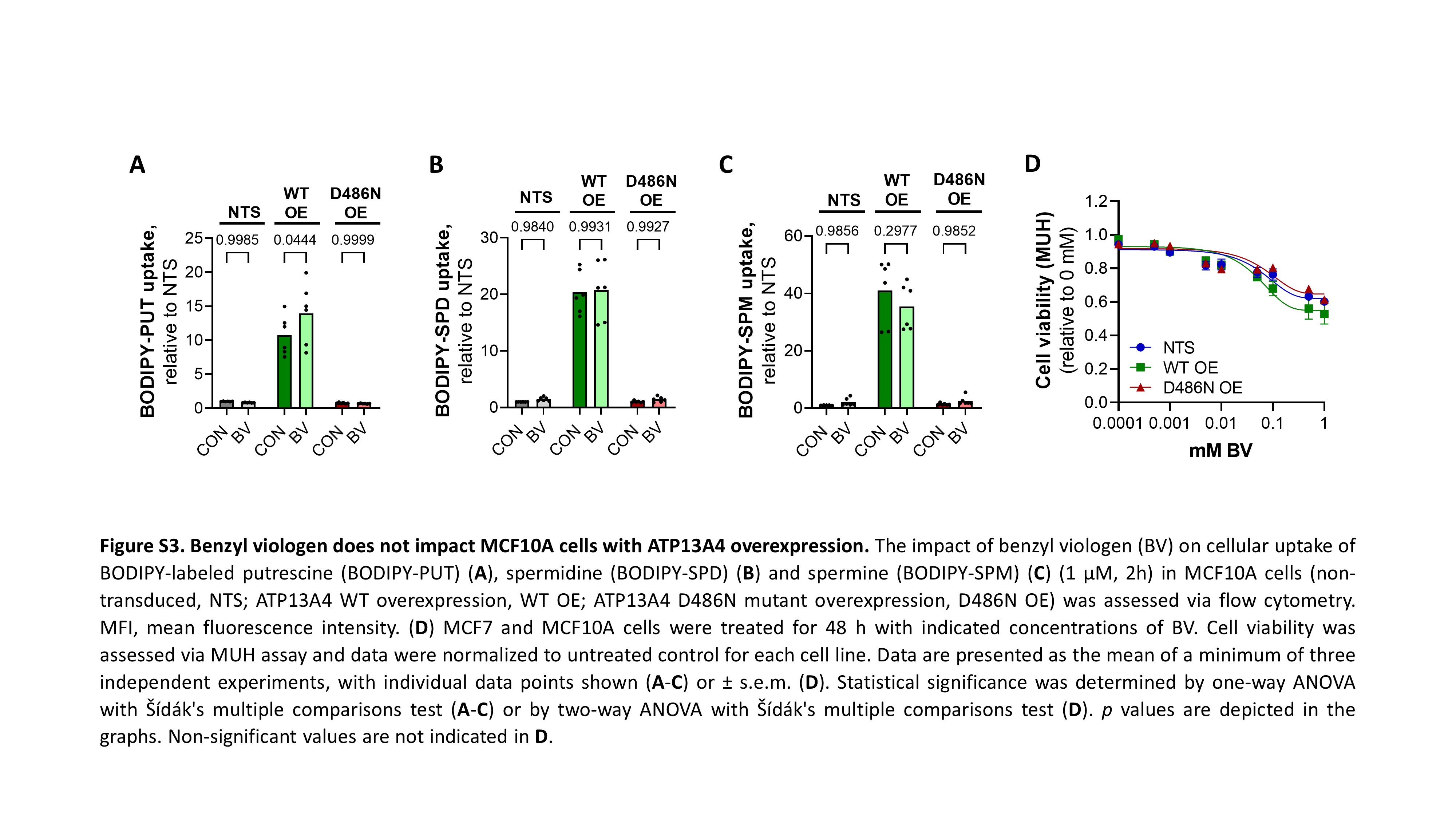

Supplement: Supplementary file 1 [file biomolecules-13-00918-s001.zip › Figure S3.JPG]

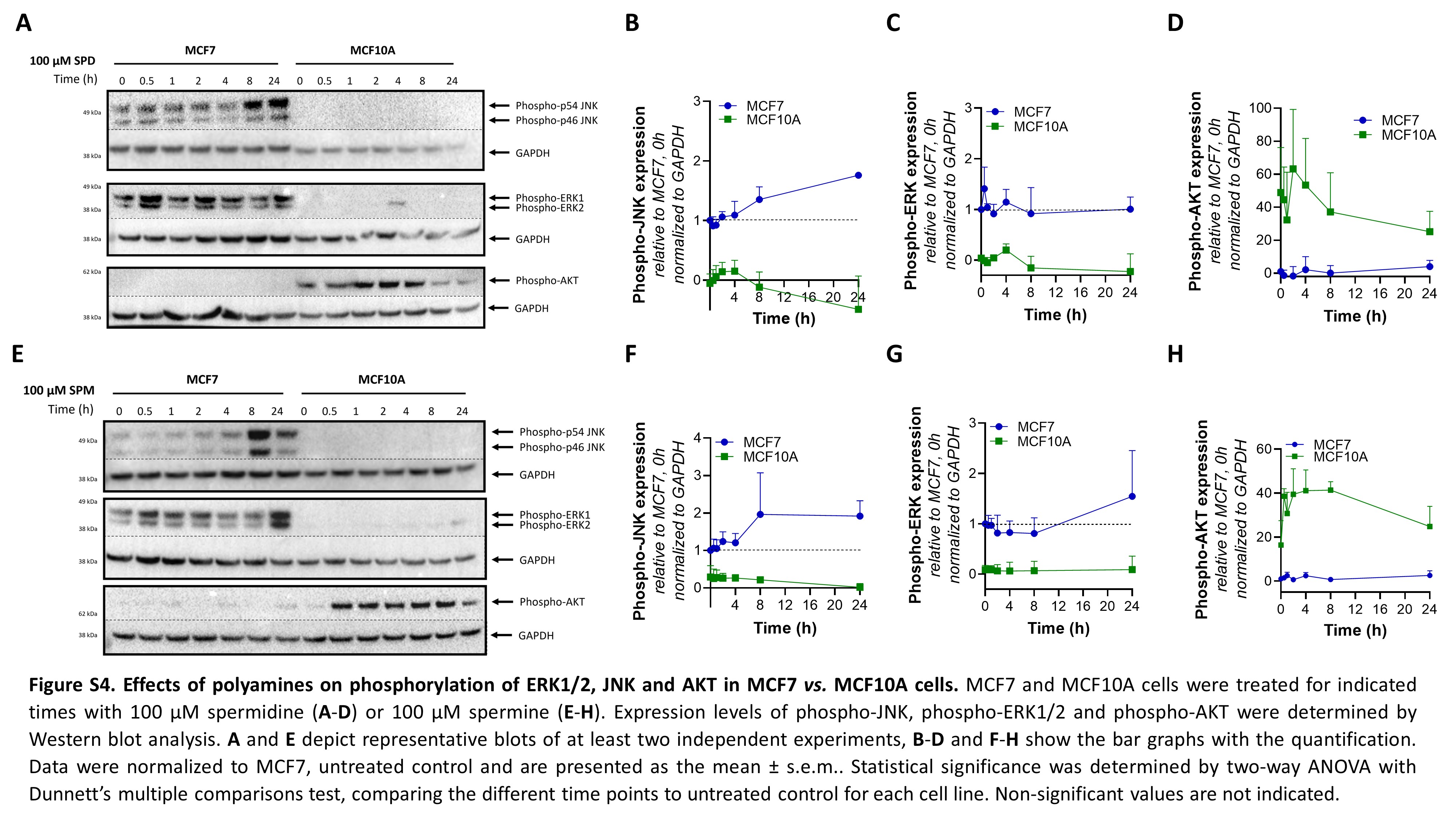

Supplement: Supplementary file 1 [file biomolecules-13-00918-s001.zip › Figure S4.JPG]
